# A case report of procedural management of an adult with morquio syndrome undergoing transcatheter aortic valve implantation

**DOI:** 10.1093/ehjcr/ytaf117

**Published:** 2025-03-07

**Authors:** Agustin Oneto, Rahul Sakhuja, Asishana Osho, Nathaniel B Langer, Matthew J Eagleton, Michael G Fitzsimons

**Affiliations:** Department of Anesthesia, Critical Care, and Pain Medicine, Massachusetts General Hospital, 55 Fruit Street, Boston, MA 02114, USA; Cardiology Division, Department of Medicine, Interventional Cardiology Section, Massachusetts General Hospital, 55 Fruit Street, Boston, MA 02114, USA; Cardiac Surgery Division, Department of Surgery, Massachusetts General Hospital, 55 Fruit Street, Boston, MA 02114, USA; Cardiac Surgery Division, Department of Surgery, Massachusetts General Hospital, 55 Fruit Street, Boston, MA 02114, USA; Vascular Surgery Division, Department of Surgery, Massachusetts General Hospital, 55 Fruit Street, Boston, MA 02114, USA; Department of Anesthesia, Critical Care, and Pain Medicine, Massachusetts General Hospital, 55 Fruit Street, Boston, MA 02114, USA

**Keywords:** Case report, Aortic valve, Genetic disorders, Inherited metabolic disorders, Valve replacement

## Abstract

**Background:**

Morquio syndrome is an autosomal recessive deficiency of *N*-acetylgalactosamine-6-sulphate, causing an accumulation of glycosaminoglycans that leads to musculoskeletal and cardiopulmonary abnormalities. We describe the management of aortic stenosis via a transcatheter aortic valve implantation (TAVI) in a patient with Morquio syndrome.

**Case summary:**

A 73-year-old woman with Morquio syndrome presented with one year of progressive dyspnoea and was found to have severe aortic stenosis on transthoracic echocardiogram. She previously underwent an attempted TAVI via transfemoral approach, which was aborted due to iliac artery dissection and occlusion. A multidisciplinary team opted for TAVI via a surgical femoral artery cut-down approach. She was last seen and recovering well three months post-procedure.

**Discussion:**

Musculoskeletal and cardiopulmonary manifestations of Morquio syndrome place patients at higher risk for procedural complications. As medical techniques improve and patients survive later into adulthood, increasing numbers will require aortic valve replacements to improve quality of life. This case highlights the challenges of aortic valve disease management in patients with Morquio syndrome, and suggests that TAVI can be safely performed using a surgical femoral cut-down approach.

**Learning points**

- Morquio syndrome (mucopolysaccharidosis IV) is an autosomal recessive deficiency of *n*-acetylgalactosamine-6-sulfate, causing an accumulation of glycosaminoglycans that leads to musculoskeletal, cardiopulmonary, and vascular abnormalities.
- The physical manifestation of Morquio syndrome in elderly patients may preclude surgical approaches to the management of valvular heart disease. Transcatheter procedures may be an option, and a multidisciplinary approach to planning and hybrid management strategies can optimize outcomes in similar patients.

## Introduction

Morquio syndrome (mucopolysaccharidosis IV) is an autosomal recessive deficiency of *N*-acetylgalactosamine-6-sulfate, causing an accumulation of glycosaminoglycans (GAGs) that leads to musculoskeletal and cardiopulmonary abnormalities.^1^ The estimated incidence of Morquio syndrome ranges from 1/71 000 to 1/500 000 births, with a mean life expectancy of 25.3 years.^2^ Musculoskeletal involvement includes short stature, pectus carinatum, macrocephaly, prognathism, short and curved limbs, and dens hypoplasia leading to atlantoaxial instability and spinal cord compression. Pulmonary involvement includes tortuous and narrowed tracheas, restrictive lung physiology due to kyphoscoliosis, and pulmonary hypertension from obstructive sleep apnea due to macroglossia, narrowed airways, and abnormal vocal folds.^3,4^ Cardiovascular involvement includes aortic and mitral valve thickening and infiltration of mucopolysaccharides in all arteries. Cardiac manifestations contribute to morbidity and can often be intervened upon. In this case report, we present a patient with Morquio syndrome and severe aortic stenosis undergoing transcatheter aortic valve implantation (TAVI), after an initial attempt at a different institution was aborted due to vascular access complications. We highlight procedural modifications that made TAVI possible for this patient with challenging anatomy.

## Summary figure

**Figure:**
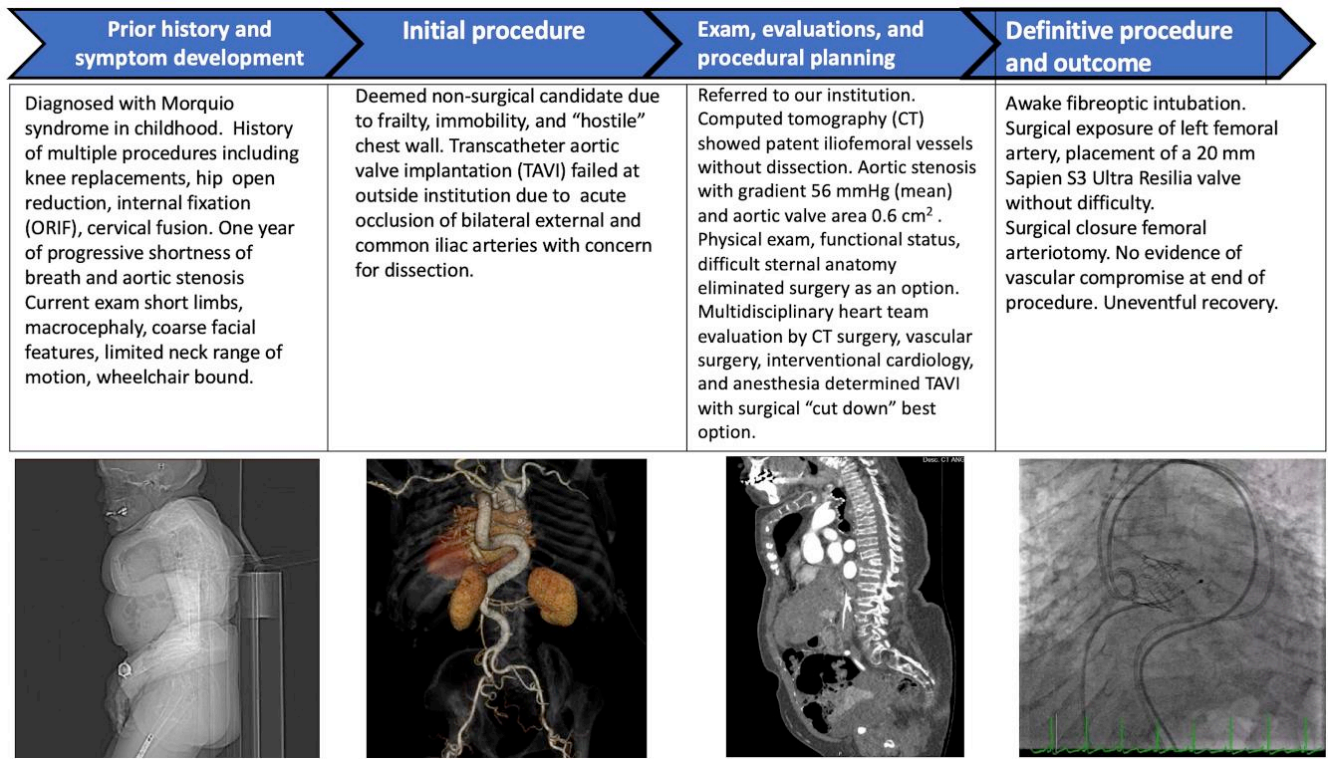


## Case presentation

A 73-year-old female with Morquio syndrome and severe aortic stenosis presented for TAVI. The patient was diagnosed with Morquio syndrome in childhood, confirmed later via molecular genetic testing. She previously underwent multiple orthopaedic surgeries under general anaesthesia several years prior, including bilateral knee replacements, hip open reduction and internal fixation, and a cervical fusion of C1-C3, and ultimately became wheelchair bound. At age 73, she reported 1 year of progressive exertional dyspnoea, dizziness, and inability to participate in essential physical rehabilitation. She was diagnosed with severe aortic stenosis. She was deemed high-surgical risk for aortic valve replacement (AVR) due to frailty, immobility, and a hostile chest wall. She underwent an attempted TAVI via transfemoral approach at a different institution. This procedure was complicated by acute occlusion of bilateral external and common iliac arteries with concern for dissection. She was treated conservatively with aspirin and anticoagulation with recanalisation of bilateral iliofemoral arteries. She was referred to our institution for further care.

**Figure 1:**
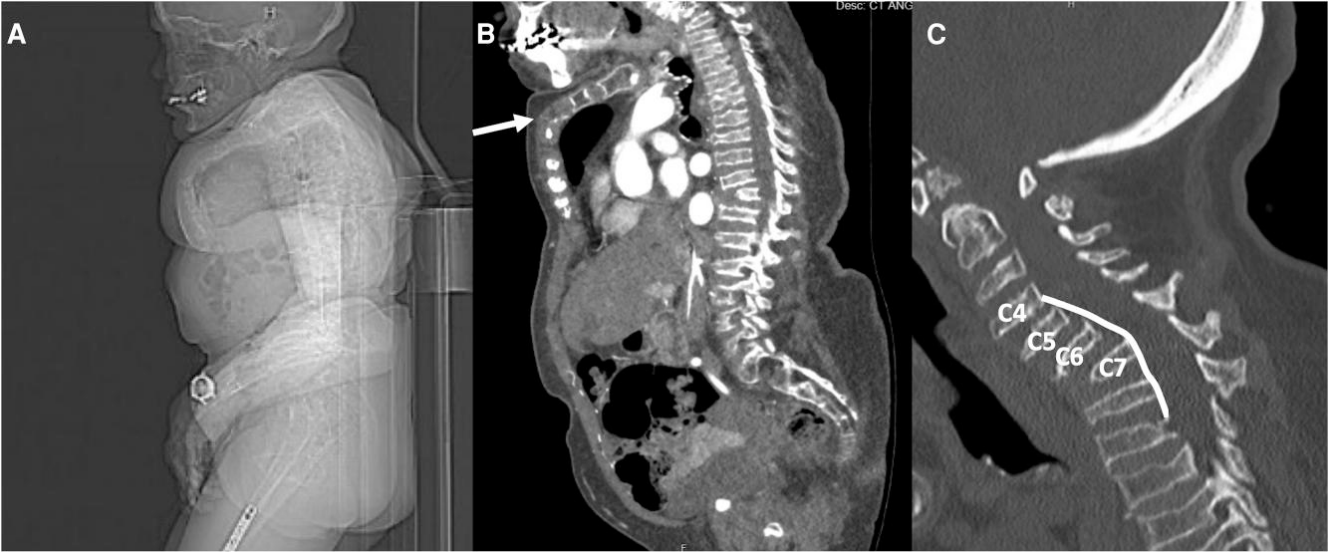
(*A*) Computer tomography scout film demonstrating short torso, neck, and sternal deformity. (*B*) Computer tomography demonstrating sternal deformity (arrow), loss of height of the vertebral bodies. (*C*) Computer tomography demonstrating cervical anterolisthesis of the fourth through seventh cervical vertebrae (C4-C7) and kyphosis (line).

**Figure 2:**
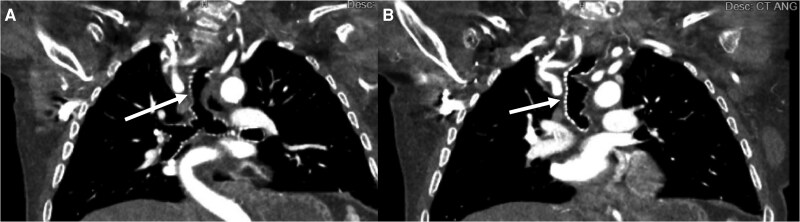
(*A*) Tortuosity of the trachea (arrow). (*B*) Angulation of the trachea (arrow).

Physical examination revealed a 39 kg, 116 cm tall female, with shortened and curved limbs, macrocephaly, and coarse facial features. Her torso and neck were short, with limited neck range of motion but a normal mouth opening (*Figure 1*). Auscultation revealed clear lungs and a systolic murmur at the right upper sternal border. Evaluation by anaesthesiology noted a short thyromental distance and Mallampati Class 3 airway. Transthoracic echocardiography (TTE) demonstrated severe aortic stenosis with a mean gradient of 56 mmHg and a valve area of 0.6 cm^2^. There was thickening of the mitral valve without restricted movement. There was no left ventricular hypertrophy. Computer tomography (CT) reconstruction revealed an aortic annulus diameter of 22.1 × 18.9 mm, circumference of 66.7 mm, and area of 3.40 cm^2^ (*Figure 2*). The iliofemoral arteries were patent without residual dissection, albeit with severe tortuosity. The minimal diameter of the right iliac system was 4.7 × 4.3 mm, and 4.9 × 4.2 mm in the left iliac system (minimal accepted iliofemoral diameter for 14 Fr TAVI ≥ 5 mm). Additionally, there was tortuosity and narrowing of the trachea related to tortuous aortic arch vasculature, with a sharp bend of 3 cm cephalad to the carina (*Figure 2*). Cardiac CT showed minimal non-obstructive coronary artery disease.

**Figure 3:**
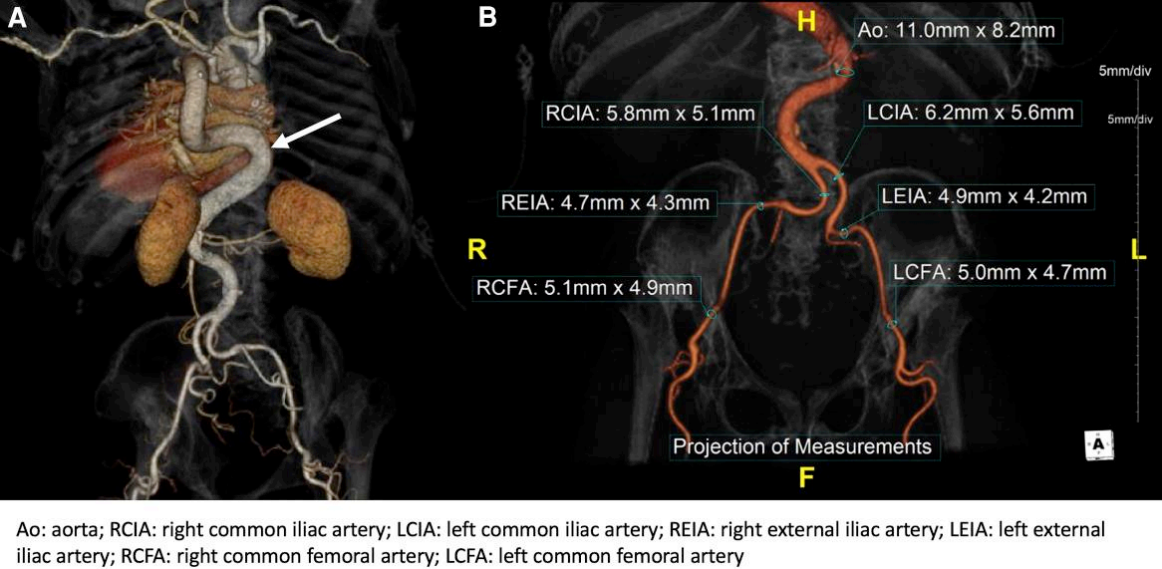
(*A*) 3D computer tomography reconstruction demonstrating tortuosity of the thoracic and abdominal aorta (arrow). (*B*) 3C computer tomography reconstruction of the abdominal aorta and iliofemoral vessels demonstrating severe tortuosity, borderline iliofemoral size, and absent underlying femoral heads bilaterally.

The patient was evaluated by cardiac surgery, who noted neck anatomy that would limit traditional sternotomy. Her sparse sternal bone raised concerns for sternal wound healing, making mini sternotomy a suboptimal approach (*Figure 1*). Given that her functional status was limited due to reliance on a wheelchair, she was deemed a poor candidate for other surgical AVR approaches such as thoracotomy, due to concerns that she would be unable to engage with the physical therapy component of post-operative recovery. Our multidisciplinary valve team deemed her optimally served by TAVI. Her short neck precluded a transcarotid approach. Her musculoskeletal chest wall abnormalities rendered any transaxillary, transaortic, or transapical approaches untenable. In collaboration with vascular surgical colleagues experienced with endovascular aortic repair, a transfemoral approach was deemed optimal given lack of vascular calcification and borderline but likely adequate sizing (*Figure 3*). Given prior percutaneous failure, lack of countertraction to successfully pass large bore sheaths, and absent femoral heads due to this patient’s syndrome, our team believed a surgical cut-down for large bore access would be more successful, and more likely to address any complications.

The patient’s past anaesthetic history suggested that ‘awake’ fibreoptic intubation was indicated. In the seated position, the airway was tropicalized with nebulized and atomized 4% lidocaine. Fentanyl and midazolam were administered to supplement topical anaesthesia. Fibreoptic laryngoscopy showed a narrowed glottic opening with redundant laryngeal tissue, angulated cords, and a large redundant epiglottis. The fibreoptic bronchoscope was advanced to the carina, and an endotracheal tube was advanced but appeared to be positioned against a wall. The airway was extubated and successfully reintubated. A transoesophageal echocardiogram probe was inserted orally.

**Figure 4:**
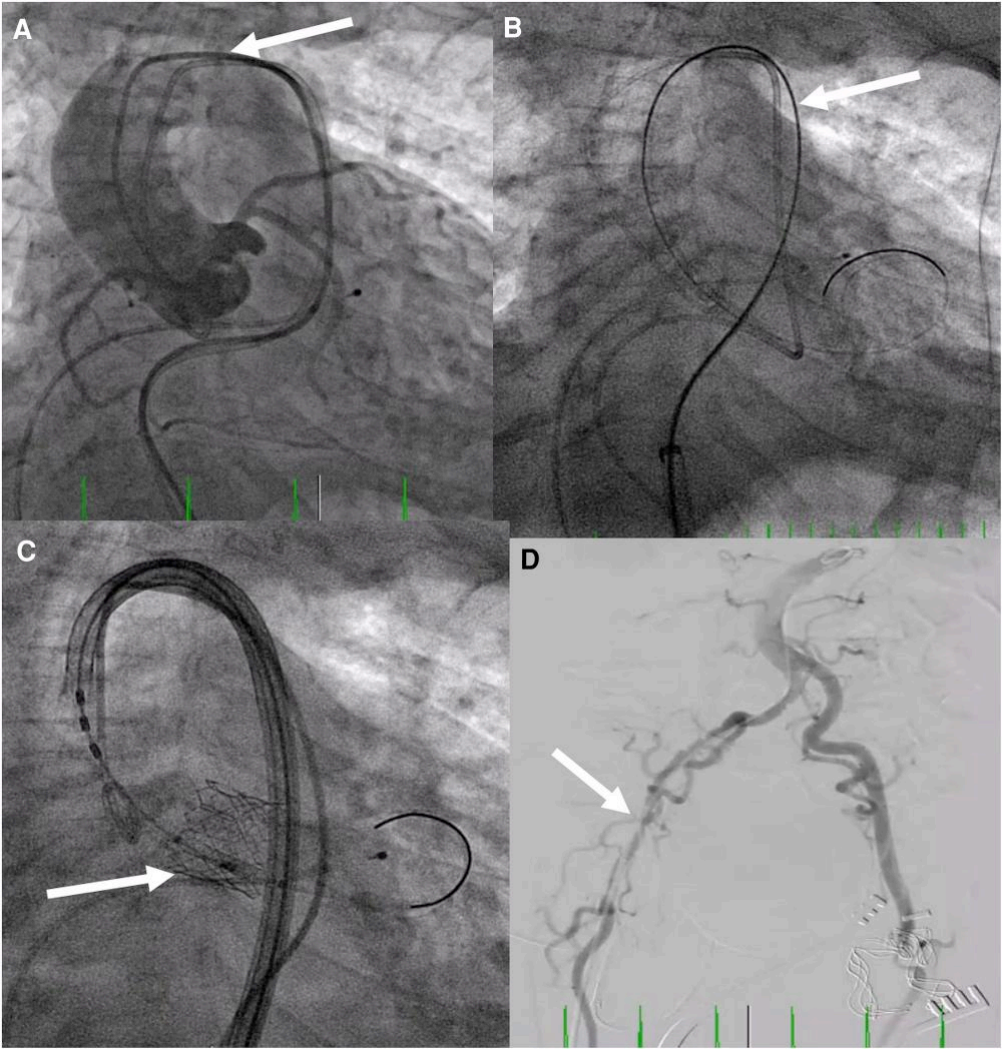
(*A*) Severe tortuosity of aortic arch (arrow) that required navigation. (*B*) Significant and important reduction in aortic arch tortuosity with introduction of Lunderquist wire (arrow) (Cook Medical, Bloomington, IN, USA). (*C*) Successful deployment of 20 mm Sapien Ultra RESILIA Edwards balloon expandable valve (Edwards Lifesciences Corporation, Irvine, CA, USA) fully deployed (arrow). (*D*) Final angiogram demonstrating patent left iliofemoral system after removal of wires and sheaths and surgical closure of arteriotomy, and pseudostenosis of right external iliac system (arrow) with just a 6F Pigtail catheter.

Due to slightly larger size and slightly less tortuosity, the left common femoral artery was surgically exposed by vascular surgery. Micropuncture access, a 0.035’ Versacore wire (Abbott Vascular, Plymouth, MN), and an initial 5F 25 cm (long) sheath were used to carefully navigate the iliofemoral arteries. The right femoral vein and right common femoral artery were cannulated using ultrasound guidance, micropuncture, and a modified Seldinger technique for intraprocedural transvenous pacing and supravalvular angiography, respectively, as per routine. Heparin was administered. The wire was exchanged for a 0.035’ (stiff) Lunderquist wire. The artery was serially dilated with dilators, and a 14 Fr expandable sheath was advanced under direct visualisation of the arteriotomy and fluoroscopic guidance without difficulty. ViperSlide™ (Abbot Laboratories, Lake County, Illinois, USA) was used to coat the dilators and the sheath and maximize lubrication of the sheath. The sheath was introduced over a stiff Lunderquist wire to maximize intralumenal support through tortuous vasculature. We opted to deploy the valve over a Lunderquist wire in the left ventricle, which significantly decreased the tortuosity in the aortic arch, facilitated valve delivery to the native annulus, and minimized movement of the valve during deployment. We paid careful attention to limit wire movement in the aorta and ventricle to minimize the risk of using this wire. We opted to use a Sapien S3 Ultra Resilia valve to take advantage of the deflectable sheath given the severe vascular tortuosity and minimize pushing against the aorta. Given the patient’s horizontal root, a balloon aortic valvuloplasty (18 × 40 mm Maxi balloon, Cordis, Miami, FL) was performed to facilitate valve delivery. Given the patient’s small stature, limited mobility, limited longevity based on natural history, borderline annular size reasonable for a 20 mm implant, and concern for vascular/annular injury with any oversizing, our team felt the risk/benefit favored implantation of a 20 mm Sapien S3 Ultra Resilia balloon expandable valve. The valve was deployed in an appropriate position without significant difficulty (*Figure 4*). The delivery system and sheath were removed in a slow and intentional manner without any evidence of vascular disruption. There was significant pseudostenosis of the right external iliac artery, which may have played a role in the acute closure during the initial TAVI attempt at another facility (*Figure 4*). The left femoral arteriotomy was primarily repaired with adequate flow by angiography, Doppler, and pulse volume recordings. Heparinisation was reversed with protamine. Maintenance anaesthesia was weaned, and the patient resumed spontaneous breathing and was extubated awake without complication.

Post-procedure lower extremity ultrasonography showed no pseudoaneurysm or dissection, and TTE showed normal bi-ventricular function, a well-seated replaced aortic valve, aortic mean gradient of 9 mmHg, and no paravalvular leak. The patient endorsed exertional dyspnoea in the first week following TAVI, but this gradually improved. Three months later she was recovering well, increasing her activity level, and working with home physical therapy.

## Discussion

Morquio syndrome (mucopolysaccharidosis IV) is an autosomal recessive deficiency of *N*-acetylgalactosamine-6-sulphate, causing accumulation of GAGs in nearly all tissues that leads to musculoskeletal and cardiopulmonary abnormalities.^1^ Although patients with Morquio syndrome have life expectancies <30 years, recent medical improvements have led to many surviving past the seventh decade of life.^5^ As more patients with Morquio syndrome survive into late adulthood, increasing numbers will need AVRs to improve quality of life. As is evident from this case, the musculoskeletal and cardiopulmonary manifestations of the condition complicate every aspect of care. Vascular GAG deposits contribute to vascular fragility, which complicates arterial access and likely contributed to the dissection that ended the patient’s first attempt at TAVI. Moreover, musculoskeletal abnormalities render high risk for surgical AVR and may preclude alternative access for TAVI.

This case demonstrates the advantages of careful pre-operative planning and the utility of awake intubation. Nearly all types of mucopolysaccharidoses are associated with anatomic abnormalities that may impact airway management, including facial and oral conditions.^6^ GAG deposits in the tongue, mandible, and laryngeal tissue increase the potential for obstructive sleep apnea and difficulty with ventilation. GAG deposits in the laryngeal tissue and trachea leads to tortuous and narrowed tracheas that pose challenges during intubation, including via fibreoptic approach.^4^ Given the high likelihood for a ‘cannot intubate, cannot ventilate’ scenario in patients with Morquio syndrome, proceeding directly to awake fibreoptic intubation with a small caliber endotracheal tube is often the safest approach to airway management. Pre-operative considerations include careful review of pulmonary function testing to guide ventilation parameters given the high rates of restrictive pulmonary disease, review of CT imaging to guide approach to fibreoptic intubation in the setting of abnormal tracheal anatomy, and review of cardiac testing to guide the choice of induction and maintenance agents. Post-operatively patients with Morquio syndrome should be extubated awake and allowed to sit up and mobilize as early as possible to minimize airway complications.

In addition to the anaesthetic considerations listed above, several procedural modifications were needed to safely perform TAVI in our patient with vascular fragility due to Morquio syndrome. The use of a surgical cut-down, stiff wires, and ViperSlide™ (Abbot Laboratories, Lake County, Illinois) to maximize lubrication of large bore dilators and sheaths was essential to safely manage tortuous vessels of borderline size. The techniques were employed to avoid prior acute closure/dissection seen during a percutaneous transfemoral arterial approach. Additionally, we performed pre-dilation balloon aortic valvuloplasty to prevent any additional resistance to crossing the valve in this patient’s horizontal aortic root. Although relative contraindications precluded surgical AVR in this case, there have been successful reports of this in younger patients with Morquio syndrome with vascular anatomy deemed unsuitable for TAVI.^7,8^ Surgical approaches, including ministernotomy and thoracotomy, should be considered in cases where TAVI is not feasible.

## Conclusion

In this case we describe a successful TAVI in a patient with Morquio syndrome, characterized by absent femoral heads and vascular fragility, using a surgical cut-down approach for vascular access. Similar procedural modifications to those posed in this paper may be necessary for patients with Morquio syndrome or other similar congenital diseases who necessitate aortic valve or other structural interventions.

## Data Availability

Data used in this paper is available to readers upon request.

## Lead author biography

Agustin Oneto is a third year resident at the Massachusetts General Hospital, undergoing training in the Department of Anesthesia, Critical Care and Pain Medicine. He is originally from Buenos Aires, Argentina and Miami, Florida. He has an undergraduate degree from the University of Chicago, and a Medical Doctorate from Harvard Medical School.

## Notes

**Consent:** The authors confirm that a written consent for submission and publication of this case report including images and text was obtained from the patient in line with COPE guidance.

**Funding:** None declared.
